# The MAR method versus the visual estimation method in predicting external blood loss: a randomized controlled study

**DOI:** 10.1038/s41598-025-16169-0

**Published:** 2025-08-24

**Authors:** Betul Akbuga Ozel, Gurkan Ozel, Elmas Burcu Mamak Ekinci, Ahmet Eren Demirtola, Gizem Sahin, Gokcen Aksoy, Ilke Yanikyurek, Mehmet Ufuk Karaaslan

**Affiliations:** 1 Department of Emergency Medicine, Ankara Yıldırım Beyazıt University Faculty of Medicine, Ankara Bilkent City Hospital, Ankara, Turkey; 2 County of Renfrew Paramedic Services, ON Renfrew, Canada; 3 Department of Computer Engineering, Baskent University Faculty of Engineering, Ankara, Turkey; 4 Baskent University Faculty of Medicine, Ankara, Turkey

**Keywords:** Clinical skill training, Emergency medicine, External blood loss, MAR method, Randomized controlled study, Visual estimation, Health care, Medical research, Signs and symptoms

## Abstract

Accurately estimating blood loss is critical for managing hemorrhagic shock in prehospital and hospital settings. The Visual Estimation (VE) method is widely used, while the MAR method proposes using a fist measurement to estimate external blood on flat surfaces. A randomized, double-blind, parallel-group, controlled, and educational study was conducted involving 140 medical and paramedic students. The study compared the accuracy and duration of blood loss estimates made with the MAR and VE methods. The secondary objective was to assess whether MAR estimates varied by participants’ height, weight, or gender. Three simulated stations featured three different, non-absorbent, flat surfaces containing 75 mL, 150 mL, or 750 mL of blood. Participants were divided into two groups (Group 1: VE method, n_1_ = 70; Group 2: MAR method, n_2_ = 70) and asked to estimate blood loss using the assigned method at each of the three stations (Station 1 to Station 3). Analytical tests evaluated absolute differences and percentage errors in both intergroup and intragroup comparisons. In the intergroup analysis, comparisons were made between Group 1 using the VE method and Group 2 using the MAR method. In the intragroup analysis, Group 1 was compared using the VE method versus the MAR method. Given the non-normality, repeated measures were analyzed using F1 LD F1 and F2 LD F1 models via nparLD in R. Improvement in estimations after the MAR method was evidenced by a reduction in errors relative to the actual blood volumes, and this was statistically significant. It also reduced outlier estimates and took longer. The MAR volume estimates were not affected by participants’ phenotypic characteristics. In conclusion, incorporating the MAR method into structured clinical skills training for external bleeding management could be beneficial.

**Trial registration**: ClinicalTrials.gov Identifier NCT06855472 (18/02/2025).

**Supplementary Information:**

The online version contains supplementary material available at 10.1038/s41598-025-16169-0.

## Introduction

Hemorrhage is the most common cause of shock in patients with polytrauma, leading to cellular hypoxia and death^1^. Physicians are frequently required to estimate blood loss from hemoptysis, epistaxis, rectal bleeding, vaginal bleeding, and traumatic injury as part of their physical examinations and ongoing evaluations^2–6^. Fluid and blood resuscitation in hemorrhagic shock involves aggressive intervention to restore lost blood volume, regain tissue perfusion, and reduce mortality^1^.

The ability to correctly estimate the amount of external blood loss is considered an important skill, as it is related to the classification and management of hemorrhagic shock by pre-hospital emergency care services, emergency departments, and surgical unit professionals. However, this ability is a relatively overlooked part of both undergraduate and postgraduate emergency medicine, paramedicine, and surgical education. At present, there is no consensus among experts in the field on how best to estimate the amount of external blood loss. Thus, most health professionals do not receive standardized, theoretical, and practically structured skills training in undergraduate and postgraduate programs. Additionally, there are only a small number of studies on external blood loss estimation in emergency medicine (hospital or pre-hospital settings)^2–11^.

Early recognition of hypovolemic shock, particularly in prehospital and hospital settings, relies mainly on visual estimation of bleeding and evaluation of vital signs^12^. However, the correlation between hemodynamic parameters (e.g., pulse, blood pressure, respiratory rate) and actual blood loss may be unreliable—especially in pediatric, geriatric, or well-compensated adult patients. In such cases, vital signs may remain within normal ranges despite significant blood loss, leading to underestimation by clinicians^12^.

Accurate estimation of external blood loss allows early recognition of probable hemorrhagic shock, rapid patient triage, and appropriate fluid resuscitation during pre-hospital care. In emergency departments, in addition to rapid and adequate fluid resuscitation, accurate estimation of external blood loss contributes to timely blood replacement, early identification of indications for surgical consultation, and early surgical interventions, if needed. Therefore, more accurate methods of blood loss estimation are essential to enable earlier detection and treatment of hypovolemia^1^.

### Visual estimation and MAR method

The Visual Estimation (VE) method is the most commonly used approach for estimating the amount of external bleeding in patients with hemorrhage^2–7^. However, previous studies have shown that paramedics and paramedic students^2–5,7^, emergency medicine specialists and residents^6,7^, other health professionals (surgeons, internal medicine specialists, nurses, technicians)^8–10^, and medical students and faculty members^11^ could not accurately predict external blood loss using this method. Using the VE method, blood loss was found to be overestimated for volumes below 300 mL and underestimated for volumes exceeding 400 mL^3^. Moreover, there was no correlation between the accuracy of the VE method and variables such as clinical experience, training and skill level, age, or gender among health students^2,11^ and health professionals^3,6–8,11^.

The limited number of studies on this subject has been criticized^10^ for several reasons, including the inadequate simulation of blood by the products used and the didactic nature of training methods, which limits knowledge transfer to clinical practice. There is currently no well-defined framework outlining the application, instructional methodologies, or assessment strategies for the VE method as either a clinical performance measure or an educational skill. This lack of standardization limits the effectiveness of training and research aimed at improving VE method accuracy. In our study, participants were not provided with any training on the use of the VE method.

Although there are no standardized application steps for estimating external blood loss using the VE method, there are some recommendations to improve its accuracy^3,7^. The visual estimation of external blood loss begins with assessing the scene, including the surface type (e.g., floor, clothing, medical equipment). Blood spread and distribution are then evaluated, considering whether it is liquid, clotted, or absorbed. Volume estimation relies on reference sizes: a palm-sized stain (~ 100 mL), an A4 paper-sized stain (250–500 mL), and a bedsheet-sized stain (> 1000 mL)^3,7^. Surface properties affect absorption and require adjustment—fabric retains blood, while non-absorbent surfaces exaggerate loss. The estimated volume should be correlated with clinical signs (e.g., blood pressure, heart rate, mental status) to assess severity^7^. While practical, visual estimation is subjective and should be verified with objective measurements when possible. In our study, participants were not provided with training on the VE method.

The MAR (Merlin, Alter, Raffel) method, first described by Merlin et al.^10^ in 2009, is recommended for estimating external blood loss on flat surfaces and does not depend on visualization. Initially, Merlin et al. were inspired by the burn area estimation method, where a patient’s palm roughly equals 1% of total body surface area^12^. Building on this concept, the researchers equated the anterior surface of a clenched fist to a certain blood volume on a flat surface^10^.

In Merlin et al.’s study, they examined the MAR method using blood volumes of 450 mL to 1,200 mL on flat surfaces (vinyl, acrylic glass, etc.), in increments of 20, 25, and 60 mL. Two researchers (males, weighing > 70 kg and height > 170 cm) each made a fist and moved their fists over the blood accumulations, with the dorsum of the fist facing upward and held 5 cm above the surface^10^. Using this technique, they recorded how many fists were required to cover each blood-bearing surface area completely. They repeated this process for each volume increment. The data obtained were then statistically analyzed. The blood surface area covered by one fist was assigned a value in milliliters. Ultimately, a two-way analysis of variance revealed that one fist equaled approximately 20 mL of blood. The results showed a correlation between the two researchers’ measurements but no significant differences in volume estimates between the two surfaces tested.

Although there are no standardized recommendations for the implementation steps of the MAR method, the application consists of two fundamental steps^10^. The first step in the MAR method is that a fist-sized blood pool is approximately equivalent to 20 mL of blood loss. In the second step, the number of fist-sized blood pools is counted, and the total estimated blood loss is calculated by multiplying the number of fists by 20 mL^10^. To enhance the effectiveness of the MAR method, the fist should be held at eye level or slightly above to fully visualize the blood pool^10^. To improve the accuracy of visual estimation, it is recommended that the fist is not held too close or too far from the blood. Generally, an assessment distance of approximately 30–50 cm is recommended^10^. In our study, the MAR method was chosen as the intervention for the experimental group. The group applying the MAR method received a 1–2 min training session before making estimations.

### Importance, objectives, and outcomes

The importance of this study lies in its distinction as, to our knowledge, the first randomized controlled trial to evaluate the accuracy of MAR method estimates by comparing two methods (VE and MAR) since the MAR method’s introduction in 2009. Furthermore, it is novel in examining whether MAR method estimates are influenced by variables such as gender, height, and weight, while also including undergraduate medical and paramedic students in the study.

The primary objective of this study was to compare blood loss estimates, estimation durations, absolute differences (between the estimated and actual blood volumes), and percent errors (calculated as the absolute difference divided by the actual blood volume and expressed as a percentage) obtained using the MAR method and VE method between randomized groups. The secondary objective was to determine whether participants’ height, weight, and gender affected estimates made using the MAR method.

The primary outcomes of the study were blood loss volume estimates (mL) and estimation durations (seconds) using the MAR method and VE method. The secondary outcomes were blood loss volume estimates (mL) and estimation times (seconds) made by participants using the MAR method based on physical variables such as height (< 170 cm, > 170 cm), weight (< 70 kg, > 70 kg), and gender (female, male).

### Hypotheses

- Hypothesis 1: The MAR method would yield more accurate blood loss estimates than the VE method on flat and non-absorbent surfaces.
- Hypothesis 2: The MAR method would result in longer blood loss estimation durations compared to the VE method.
- Hypothesis 3: Demographic variables, such as gender, height, and weight, would affect blood loss volume estimates using the MAR method.
- Hypothesis 4: Demographic variables, such as gender, height, and weight, would affect estimation durations in blood loss volume assessments using the MAR method.

## Methods and materials

### Ethical declarations

This study was approved by the Ethics Committee and Research Council of Başkent University Medical and Health Sciences (Project No: KA17/92) and was planned based on the CONSORT 2010 Checklist for a parallel-group randomized controlled trial. The study was conducted between March 30, 2017, and May 10, 2017. Informed consent was obtained from all participants. After their consent was obtained, their gender, height, and weight information were collected from the Başkent University Health Unit. The study was conducted in accordance with the principles of the Declaration of Helsinki. The study was registered as a clinical trial on 18/02/2025. The trial registration number (ClinicalTrials.gov Identifier) is NCT06855472.

### Study population

A total of 237 second-year medical students and second-year paramedic students of Başkent University were invited to participate in this study. Students in the medicine and paramedic departments were regarded as similar groups in terms of academic year, age, educational background, lack of training in estimating external blood loss in their programs, and absence of clinical experience.

### Sample size, effect, and power

The sample size was determined using G-Power 3.1 statistical software^13^. According to Cohen^14^, effect size is a quantitative measure of the magnitude of the experimental effect. An effect size of less than 0.1 is considered small, values between 0.3 and 0.5 indicate a medium effect, and values greater than 0.5 represent a large effect^14^.

In the absence of prior data, a medium effect size (Cohen’s d = 0.5) is often used as a conventional estimate for sample size calculation, based on Cohen’s guidelines^14^. Since there was no reported analysis of effect size in the reference article^10^, our study aimed to determine a sample size that would provide a medium effect size.

Based on the research hypotheses, the required sample size for each group was calculated as approximately 60 participants, considering an alpha level (α) of 0.05, an effect size of 0.5, and a statistical power of 0.85. Consequently, the required sample size was determined to be 60 participants per group, resulting in a total of 120 participants. To account for potential dropouts, 20 additional participants were included, resulting in a final sample size of 70 participants per group, and 140 participants in total.

### Study design

**Fig. 1:**
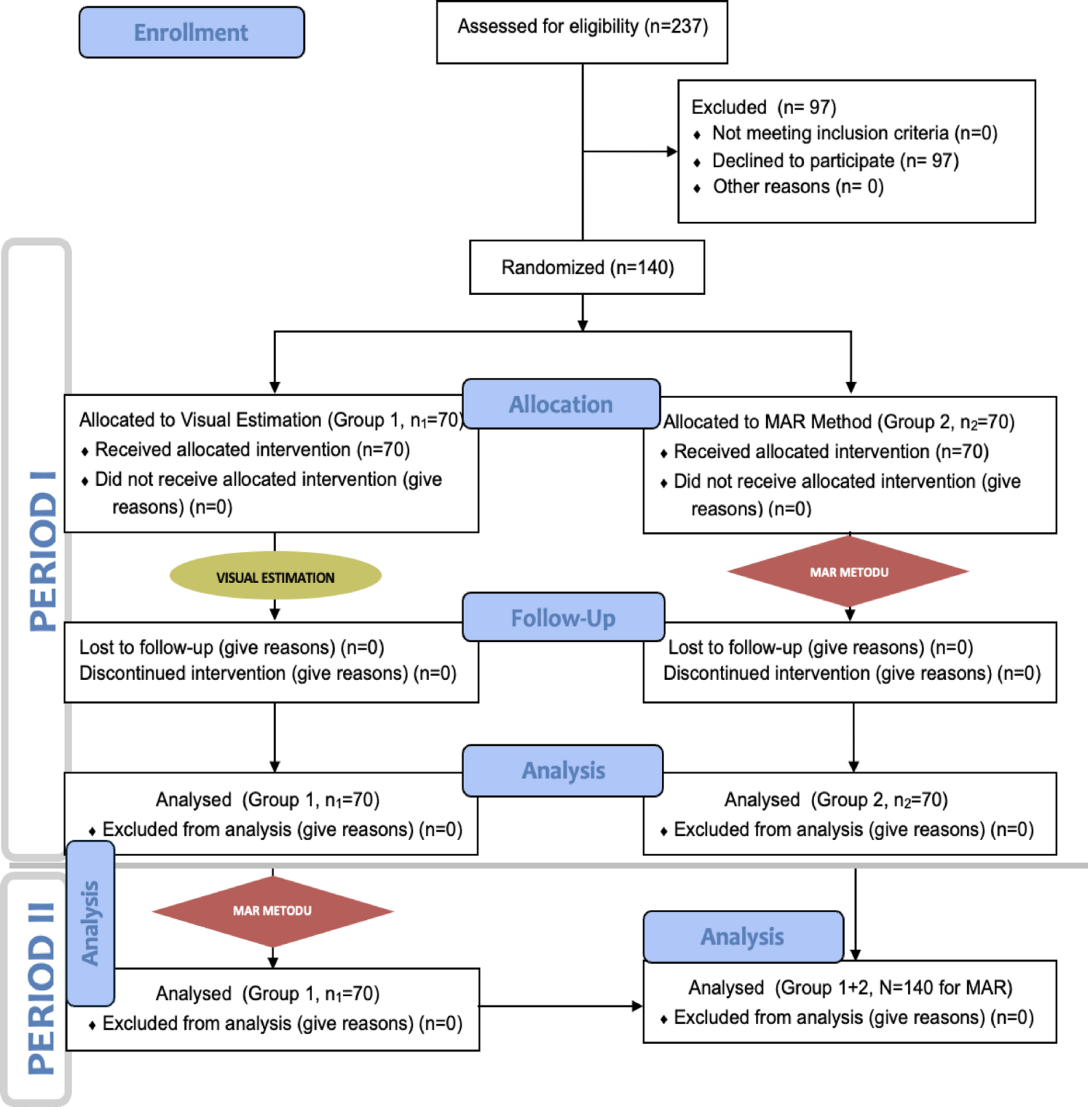
Consort flow diagram.

The study was designed as a single center, double-blind, randomized, parallel-group, controlled, and clinical skill-focused educational study. Figure 1 shows the study’s flow diagram arranged according to the CONSORT 2010 protocol (Fig. 1).

### Inclusion criteria and randomization

The pre-randomization inclusion criteria were being a second-year paramedic student or a second-year medical student, having received no theoretical or practical training in predicting external blood loss, and agreeing to participate in the study.

Of the 237 eligible participants, 140 met the inclusion criteria and consented to participate. These 140 participants were randomized into two equal groups of 70 each using the block randomization method with a 1:1 allocation ratio. The randomization process was conducted through the online service https://researchrandomizer.com. The control (Group 1, VE) and experimental (Group 2, MAR) groups were assigned by blind selection. To ensure allocation concealment, both randomization and group assignment were carried out by an independent researcher before the trial began. The three faculty researchers who prepared the stations knew the actual blood volumes at each station, whereas the participants and three different study investigators who recorded participant data and estimates were blinded to this information.

### Setting

For this study, the Professional Skills Laboratories of Başkent University’s Faculty of Medicine were utilized. Scenarios were set up at three different stations located in separate corners of a large room. Each station was isolated from the others using room dividers and had separate entrance and exit doors. Participants who completed their estimations were directed to leave through the exit door to avoid encountering participants who had not yet completed their tasks.

Two faculty researchers prepared three different simulated environments and scenarios at three stations. Three faculty researchers oversaw the orientation training for investigators and participants at the beginning of the study and coordinated activities at each station. Additionally, three medical student investigators were assigned to each station and recorded blood volume estimates and estimation times.

A synthetic blood product **(**Ben Nye Stage Blood**)** was used to augment fidelity in the simulated blood loss scenarios. Ben Nye Stage Blood is a corn syrup-based liquid designed for theatrical and special effects use. It has a medium viscosity that mimics the flow of fresh blood. Its bright red color resembles the appearance of real blood. Additionally, it is mint-flavored and safe for use in the mouth^15^.

The reflectivity characteristics of surfaces such as acrylic, wood, and vinyl can affect the accuracy of visual estimation. Features of wooden surfaces such as color, grain pattern, and gloss significantly influence human visual perception. In particular, gloss determines a surface’s ability to reflect light, which directly impacts how the surface is perceived. Wooden surfaces have a low to medium level of reflectivity^16^. Acrylic surfaces have a high level of reflectivity (glossy). They reflect light well, which can make dark-colored fluids like blood appear reduced in quantity and less defined in terms of edges. Vinyl surfaces have a medium to high level of reflectivity^17^. Vinyl typically has a smooth and glossy surface. Especially “glossy vinyl” types reflect light strongly, which can cause glare on blood stains and pooling and distort the perception of color and boundaries^17^. On the other hand, matte or satin vinyl has moderate reflectivity^17^.

In accordance with the design principles of the MAR method^10^, non-absorbent, flat surfaces were used at all three stations in this study. In Station 1, the scenario involved a high reflective acrylic (plexiglass) surface with 75 ml of blood. In Station 2, a medium reflective matte varnished wood surface was used with 150 ml of blood. In Station 3, a medium reflective satin vinyl surface was used with 750 ml of blood.

### Interventions and flow of the study

The student investigators were given a 20-minute orientation before the research about the study purpose, procedures, data form usage, and use of a chronometer. Investigators were instructed to record data concurrently as estimations were made. Neither the participants nor the investigators were aware of the actual blood volumes (double-blind).

At the study’s start, Group 1 and Group 2 received a 10-minute briefing, which included signing consent forms, explaining the study’s purpose, and introducing the simulation lab and faculty researchers.

To improve clarity in presenting the study process, the research was structured into two distinct periods. In Fig. 1, Period I includes the interventions and analyses conducted between the study’s randomization phase and the horizontal dividing line. Period II encompasses the interventions and analyses depicted below that line.

Period I was designed to obtain the primary outcomes based on the study’s primary objectives. During this phase, Group 1 first estimated blood loss using the VE method at all three stations (Stations 1 to 3). Simultaneously, participants in Group 2 received 1–2 min of training on the MAR method from an investigator in a separate room, away from the stations. After training, Group 2 participants entered the main room one at a time and completed blood loss estimations using the MAR method at each of the three stations. At each station, different investigators recorded the blood volume estimates and the time taken by Group 1 (using VE, n_1_ = 70) and Group 2 (using MAR, n_2_ = 70), providing data for intergroup analysis (Fig. 1).

Period II was conducted to obtain the secondary outcomes, in line with the study’s secondary objectives. The same investigator who recorded Group 1’s VE estimates in Period I provided a 1–2 min training session on the MAR method to Group 1 in a separate room. After training, Group 1 participants entered the main room one at a time and re-estimated blood loss at Stations 1 to 3 using the MAR method. As in Period I, investigators at each station recorded the blood volume estimates and estimation times. Since Group 1’s VE data had already been collected in Period I, the VE and MAR data for Group 1 (n_1_ = 70) were compared as intragroup data for secondary analysis (Fig. 1).

To obtain secondary outcomes based on the secondary aims of the study, the authors combined and evaluated the data of all 140 participants (n1:70 + n2:70) who used the MAR method (Group 2 in Period I and Group 1 in Period II) (Fig. 1). These data were analyzed to determine whether gender, height, or weight influenced estimation volumes or times using the MAR method.

### Data collection

A data collection form was created. The first section recorded demographic information. The second section included a table for estimates and estimation durations. Each participant had 60 s to complete their estimations. A chronometer was used. Volume estimates were recorded in milliliters (ml), and time in seconds (sec).

### Outcome measures

Estimation times and raw blood volume estimates were used to evaluate and compare characteristics across intergroup and intragroup analyses. Absolute differences and percent errors were calculated to assess accuracy—advantages not possible using only raw means or medians.

Deviations from actual volume (i.e., volume estimation errors) were calculated as the absolute differences between the estimated and actual blood volumes. They were calculated as |Estimated blood volume – Actual blood volume|. Mean, standard deviation, median, and interquartile range were then calculated to assess estimation precision.

Another parameter calculated to visualize the deviations from actual volume was the percent error. Percent error compares the estimated blood volume to the actual blood volume and expresses the difference between them as a percentage. This statistic allows us to understand the size of the error relative to the actual blood volume. Percent errors were calculated for each participant by dividing each absolute difference by the actual blood volume (75 ml, 150 ml, or 750 ml) and multiplying the result by 100. (Percent error = Estimated blood volume − Actual blood volume)/Actual blood volume × 100^10^.

### Statistical analysis

Data were analyzed using SPSS version 25.0 for descriptive statistics. These are presented as frequencies, percentages, minimum–maximum values, medians, interquartile ranges, means, and standard deviations. The assumption of normality was assessed using histograms and Q–Q plots, as well as the Kolmogorov–Smirnov test. The Kolmogorov–Smirnov test results indicated a violation of the normality assumption (Table 1). The histograms and Q–Q plots generated to visualize the distributional characteristics of the data presented in Appendix A, B (Supplementary Material). These graphical analyses also revealed deviations from a normal distribution. Based on the results of the normality test and the observed distributional patterns, the assumptions of normality were not met. Since the variables—*volume estimation*,* estimation duration*,* and percent error*—did not follow a normal distribution, nonparametric methods were employed for repeated measures at all stages of the study. All inferential statistical analyses were conducted using the F1 LD F1 and F2 LD F1 models via the *nparLD* package in the R programming language^18^.

**Table 1:** Evaluating the normality assumption. p* values determined by Kolmogorov-Simirnov normality test: <0.001 indicates p-values ​​less than 0.001.

		Estimated volume (ml)				Estimation duration (sec)					
Sitation	Statistics	VE method p^*^		MAR method	p^*^	VE method	p^*^		MAR method	p^*^	
Sitation 1 (75 ml)	Median (Range)	145 (10–500)	< 0.001	100 (50–200)	0.028	7.4 (1.1–37.4)	0.006		8.6 (3.3–20.2)	0.055	
	Mean (SD)	157.3 (130.3)		106.6 (31.2)		9.4 (6.8)			9.1 (3.9)		
	Skewness	0,9		0.3		2.1			0.9		
Sitation 2 (150 ml)	Median (Range)	200 (10-1100)	< 0.001	240 (120–640)	< 0.001	6.6 (1.5–28.4)	< 0.001		13.5 (3.6–32.7)	0.023	
	Mean (SD)	273.1 (241)		255.2 (98.3)		7.7 (5.2)			14.4 (6.5)		
	Skewness	1.4		1.7		1.9			0.80		
Sitation 3 (750 ml)	Median (Range)	525 (15-3000)	< 0.001	600 (290–1700)	0.005	7.4 (1-36.8)	< 0.001		31.1 (5.1–67)	0.028	
	Mean (SD)	707.5 (638.2)		692.1 (312.4)		9.4 (6.9)			34.8 (15.9)		
	Skewness	1.66		1.1		1.8			0.35		

For Hypotheses 1 and 2, both intergroup and intragroup analyses were conducted. Intergroup comparisons of measurement and error statistics across stations were made between Group 1 (VE in Period I, n_1_ = 70) and Group 2 (MAR in Period I, n_2_ = 70). For these intergroup analyses, the F1 LD F1 model—a nonparametric alternative to Repeated Measures Mixed ANOVA—was applied, as different participants from different groups made repeated estimations at three stations, each using a different method. Intragroup comparisons of repeated measures and error statistics were also conducted within Group 1 (who completed VE in Period I and MAR in Period II, n_1_ = 70). For intragroup analyses, the F2 LD F1 model—a nonparametric equivalent of Repeated Measures Two-way ANOVA—was used because participants from the same group performed repeated estimations at three stations using two distinct methods across two time periods. For Hypotheses 3 and 4, differences in primary outcome measures between dichotomous demographic groups—gender (female vs. male), weight (< 70 kg vs. ≥70 kg), and height (< 170 cm vs. ≥170 cm)—were examined among all users of the MAR method (Group 1 performing MAR in Period II, n_1_ = 70, and Group 2 performing MAR in Period I, n_2_ = 70). For these analyses, the F1 LD F1 model were again applied to evaluate differences in repeated estimations made with the MAR method at three stations by participants grouped into two categories based on gender, weight, and height.

In each application of the F1 LD F1 and F2 LD F1 models, the main effects (e.g., group, method, station) and interaction effects (e.g., group × station, group × method, weight × station) were examined. For each effect, the test statistic, degrees of freedom, and p-value were calculated. Subsequently, pairwise comparisons within each station were performed using the Bonferroni Correction. A p-value < 0.05 was considered statistically significant.

## Results

The majority of participants in the study group were female, comprising 55.7% (78/140). The average age of the participants was 19.7 (1.85) years. Table 2 shows the demographic characteristics of the study group (Table 2).

**Table 2:** Demographic characteristics of the study group.

Demographic features		Number (*n*)	Percentage (%)
Group	Group 1 Group 2	70 70	50 50
School	Paramedic Medicine	74 66	52.9 47.1
Age	< 20 ≥ 20 y	75 65	53.6 46.4
Gender	Female Male	78 62	55.7 44.3
Weight	< 70 kg ≥ 70 kg	90 50	64.3 35.7
Height	< 170 cm ≥ 170 cm	63 77	45 55

**Fig. 2:**
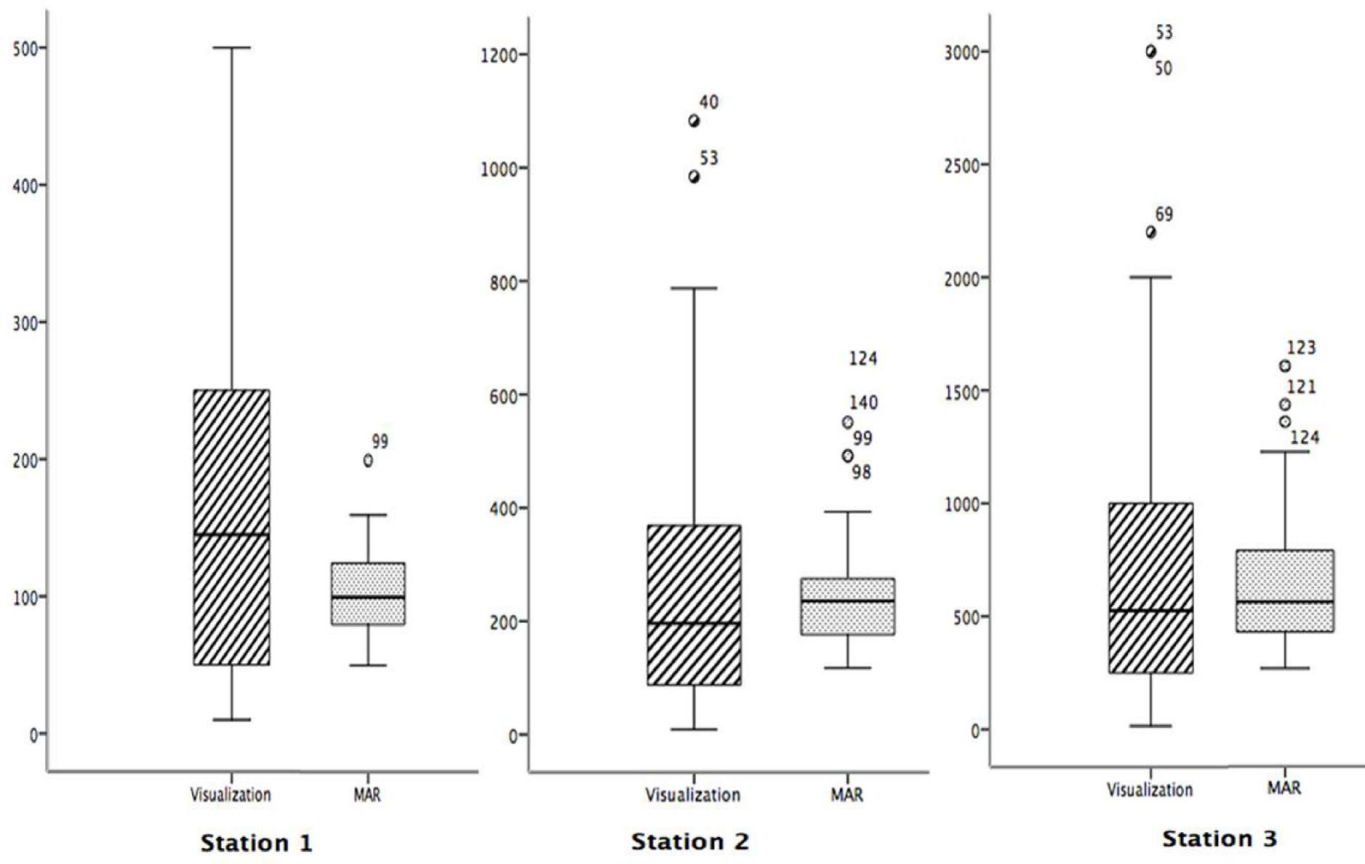
Box plot graph of volume estimates between the visual estimation and MAR groups.

In Period I, according to the intergroup raw blood estimations, the MAR method more accurately predicted actual blood loss than the VE method at stations 1 and 2 (75 ml and 150 ml, respectively). At station 3 (750 ml), both the VE and MAR methods underestimated the simulated blood volumes. There was no statistically significant difference between the volume estimations made using the VE method in Group 1 and the MAR method in Group 2 at the three stations (*p* > 0.05; F1 LD F1 model, using the *nparLD* package in R) (Table 3). At this point, a box plot graph was created to visualize the effect of both methods used by VE and MAR groups on blood loss volume estimations (Fig. 2). There was no statistically significant difference between the estimation durations using the VE and MAR methods at Station 1 (*p* = 0.5; F1 LD F1). However, at stations 2 and 3, the MAR method resulted in longer estimation durations compared to the visual method, and these differences were statistically significant (*p* = 0.001; F1 LD F1) (Table 3).

**Table 3:** Volume estimates and Estimation duration statistics between group 1 (VE in period I, n_1_ = 70) and group 2 (MAR in period I, n_2_ = 70).

		Estimated volume (ml)			Estimation duration (sec)		
Sitation	Statistics	VE method	MAR method	p^*^	VE method	MAR method	p^*^
Sitation 1 (75 ml)	Median (IQR)	145 (50–200)	100 (80-126.5)	0.31	7.4 (4.7–12.4)	8.6 (6.1–11.5)	0.5
Acrylic surface	Mean (SD)	157.3 (130.3)	106.6 (31.2)		9.4 (6.8)	9.1 (3.9)	
Sitation 2 (150 ml)	Median (IQR)	200 (87.5-381.3)	240 (180–280)	0.18	6.6 (4-9.9)	13.5 (9.5–18.3)	0.001
V-wood surface	Mean (SD)	273.1 (241)	255.2 (98.3)		7.7 (5.2)	14.4 (6.5)	
Sitation 3 (750 ml)	Median (IQR)	525 (250–1000)	600 (457.5-842.5)	0.15	7.4 (5-11.5)	31.1 (22-47.6)	0.001
Vinyl surface	Mean (SD)	707.5 (638.2)	692.1 (312.4)		9.4 (6.9)	34.8 (15.9)	
F1 LD F1 Tstat-df-p	Group	1.661 1.000 p (0.197)			Group	84.767 1.000	p (< 0.001)
	Station	747.210 1.584 p (< 0.001)			Station	65.221 1.929	p (< 0.001)
	Group x Station	35.901 1.584 p (< 0.001)			Group x Station	64.899 1.929	p (< 0.001)

For intergroup analyses, P* values were determined using F1 LD F1 model via the *NparLD* package in the R programming language. Pairwise comparisons within each station were performed using the Bonferroni Correction. Data were considered statistically significant at (*p* < 0.05). V-wood surface: Varnished-wood surface. VE method: Visual Estimation method. MAR method: Merlin, Alter, Raffel method. SD: Standard Deviation. IQR: Interquartile Range. Tstat-df-p: Test statistic-degrees of freedom-p value.

In the intragroup analyses of blood volume estimations made using the VE and then MAR methods within Group 1, the MAR method yielded estimations closer to actual blood loss amounts at Station 1 when compared to the VE method, and the observed relationship was statistically significant (*p* = 0.006; F2 LD F1 model, using the *nparLD* package in R) (Table 4). However, there was no statistically significant relationship between blood volume estimations made using the VE and MAR methods and the actual blood loss amounts at Stations 2 and 3, respectively (*p* = 0.62; *p* = 0.79; F2 LD F1). Additionally, there was no statistically significant difference in the estimation durations made using the VE and MAR methods at Station 1 (*p* = 0.31, F2 LD F1). However, at Stations 2 and 3, respectively, the MAR method resulted in longer estimation durations compared to the VE method, and the difference between the two methods was statistically significant (*p* = 0.001; *p* = 0.001, F2 LD F1) (Table 4).

**Table 4:** Volume estimates and Estimation duration statistics within group 1 (who performed VE in period I and then MAR in period II, n_1_ = 70).).

		Estimated volume (ml)			Estimation duration (sec)		
Sitation	Statistics	VE method	MAR method	p^*^	VE method	MAR method	p^*^
Sitation 1 (75 ml)	Median (IQR)	145 (50–250)	100 (90–120)	0.006	7.4 (4.7–12.4)	8.8 (6.3–11.9)	0.31
Acrylic surface	Mean (SD)	157.3 (130.3)	102.8 (25.7)		9.4 (6.8)	9.9 (5.6)	
Sitation 2 (150 ml)	Median (IQR)	200 (90–375)	250 (200–280)	0.62	6.6 (4.0-9.9)	13.1 (10.9–18.1)	0.001
V-wood surface	Mean (SD)	273.1 (240.9)	256.1 (62.9)		7.7 (5.2)	15.1 (6.7)	
Sitation 3 (750 ml)	Median (IQR)	525 (250–1000)	600 (500–800)	0.79	7.4 (4.9–11.2)	35.0 (24.1–50.6)	0.001
Vinyl surface	Mean (SD)	707.5 (638.2)	667.1 (260)		9.4 (6.9)	36.7 (14.6)	
F2 LD F1 Tstat-df-p	Group	1.818 1.000 p (0.178)			Group	145.824 1.000 p (< 0.001)	
	Station	675.344 1.502 p (< 0.001)			Station	63.923 1.887 p (< 0.001)	
	Group x Station	37.283 1.489 p (< 0.001)			Group x station	77.938 1.935 p (< 0.001)	

For intragroup analyses, P* values were determined using F2 LD F1 model via the *NparLD* package in the R programming language. Pairwise comparisons within each station were performed using the Bonferroni Correction. Data were considered statistically significant at (*p* < 0.05). V-wood surface: Varnished-wood surface. VE method: Visual Estimation method. MAR method: Merlin, Alter, Raffel method. SD: Standard Deviation. IQR: Interquartile Range. Tstat-df-p: Test statistic-degrees of freedom-p value.

The differences between actual blood volume and estimated values were statistically analyzed for both intergroup and intragroup comparisons (Tables 5 and 6, respectively). The F1 LD F1 model via the *nparLD* package in the R programming language was applied to assess intergroup measurements that did not follow a normal distribution (Table 5). Between methods, reduction percentages were calculated for the mean percent errors and for the interquartile ranges (IQRs) of the deviations from actual volume loss (i.e., errors). [Reduction percentage = ((VE value − MAR value)/VE value) × 100]. Following the application of the MAR method, the median of errors at Station 1 decreased from 70 (150) to 25 (36.3) (*p* = 0.001; F1 LD F1), resulting in a significant 67.5% reduction in percent error and a 75% decline in the interquartile range (IQR) of errors. At Station 2, the median of differences from actual volume loss decreased from 100 (181.3) to 90 (100) (*p* = 0.057; F1 LD F1), with a 41.2% reduction in percent error and a 51.6% decrease in the IQR of differences from actual volume loss. Similarly, at Station 3, the median of errors reduced from 450 (400) to 240 (252.5) (*p* = 0.001; F1 LD F1), with percentage error and IQR declining by 48.4% and 53.3%, respectively (Table 5).

**Table 5:** Error statistics of volume estimates and Estimation duration statistics between group 1 (VE in period I, n_1_ = 70) and group 2 (MAR in period I, n_2_ = 70).

Sitation	Error statistics	Errors in volume estimations		p^*^
		VE method	MAR method	
Sitation 1 (75 ml)	Mean difference (SD)	110.1 (107.5)	35.8 (26.3)	
	Median difference (IQR)	70.0 (150.0)	25.0 (36.3)	0.001
	Mean percent error (SD)	146.8 (143.3)	47.7 (35.1)	
Sitation 2 (150 ml)	Mean difference (SD)	181.5 (200.1)	106.6 (96.8)	
	Median difference (IQR)	100.0 (181.3)	90.0 (100.0)	0.047
	Mean Percent Error (SD)	121.0 (133.4)	71.1 (64.5)	
Sitation 3 (750 ml)	Mean difference (SD)	492.5 (403.9)	254.1 (188.3)	
	Median difference (IQR)	450.0 (400.0)	240.0 (252.5)	0.001
	Mean percent error (SD)	65.7 (53.8)	33.9 (25.1)	
F1 LD F1 Tstat-df-p	Method	33.598 1.000 p (< 0.001)		
	Station	14.915 1.608. p (< 0.001)		
	Method x station	3.877 1.608 p (0.029)		

For intergroup analyses, P* values were determined using F1 LD F1 model via the *NparLD* package in the R programming language. Pairwise comparisons within each station were performed using the Bonferroni Correction. Data were considered statistically significant at (*p* < 0.05). VE method: Visual Estimation method. MAR method: Merlin, Alter, Raffel method. SD: Standard Deviation. IQR: Interquartile Range. Median difference: Median of absolute differences between each estimation and the actual blood volume. Percent error: absolute difference/actual blood volume x 100. Tstat-df-p: Test statistic-degrees of freedom-p value.

**Table 6:** Error statistics of volume estimates and Estimation duration statistics within group 1 (who performed VE in period I and then MAR in period II, n_1_ = 70).).

Sitation	Error statistics	Errors in volume estimations		*p* ^*^
		VE method	MAR method	
Sitation 1 (75 ml)	Mean difference (SD)	110.1 (107.5)	32.0 (20.1)	
	Median difference (IQR)	70.0 (150.0)	25.0 (30.0)	0.001
	Mean percent error (SD)	146.8 (143.3)	42.7 (26.8)	
Sitation 2 (150 ml)	Mean difference (SD)	181.5 (200.1)	106.4 (62.5)	
	Median difference (IQR)	100.0 (181.3)	100.0 (85.0)	0.009
	Mean percent error (SD)	121.0 (133.4)	71.0 (41.7)	
Sitation 3 (750 ml)	Mean difference (SD)	492.5 (403.9)	228.9 (146.4)	
	Median difference (IQR)	450.0 (400.0)	240.0 (190.0)	0.001
	Mean percent error (SD)	65.7 (53.8)	30.5 (19.5)	
F2 LD F1 Tstat-df-p	Method	51.319 1.000 p (< 0.001)		
	Station	21.911 1.571. p (< 0.001)		
	Method x station	10.114 1.642. p (< 0.001)		

For intragroup analyses, P* values were determined using F2 LD F1 model via the *NparLD* package in the R programming Language pairwise comparisons within each station were performed using the Bonferroni Correction. Data were considered statistically significant at (*p* < 0.05). VE method: Visual Estimation method. MAR method: Merlin, Alter, Raffel method. SD: Standard Deviation. IQR: Interquartile Range. Median difference: Median of absolute differences between each estimation and the actual blood volume. Percent error: Absolute difference/actual blood volumex100. Tstat-df-p: Test statistic-degrees of freedom-p value.

The F2 LD F1 model via the *nparLD* package in the R programming language was used to evaluate intragroup measurements (Table 6). After implementing the MAR method, the median of differences from actual volume at Station 1 decreased from 70 (150) to 25 (30) (*p* = 0.001; F2 LD F1), corresponding to a 70.9% reduction in percent error and an 81.2% decrease in the IQR of errors. At Station 2, the median of errors declined from 100 (181.3) to 100 (85) (*p* = 0.009; F2 LD F1), accompanied by a 41.3% reduction in percent error and a 68.7% decrease in the IQR of errors. At Station 3, the median of errors was reduced from 450 (400) to 240 (190) (*p* = 0.001; F2 LD F1), with a 53.5% decrease in percentage error and a 63.7% reduction in the IQR of errors (Table 6).

In Period II, during which secondary outcome data were collected, Group 1 (n_1_ = 70) —having previously estimated blood loss using the VE method—completed the same task using the MAR method. These MAR data (Group 1, n_1_ = 70) were then combined with the MAR data from Group 2 (n_2_ = 70), collected during Period I (Fig. 1). The resulting dataset (*N* = 140) was analyzed across all three stations (75 mL, 150 mL, and 750 mL) to determine whether MAR-based estimates and durations were influenced by participants’ height, weight, or gender (Table 7). There was no statistically significant relationship between blood loss estimations using the MAR method and gender, height, and weight variables at any of the stations (*p* > 0.05; F1 LD F1 model via the *nparLD* package in the R programming language) (Table 7). Estimation durations at station 1 using the MAR method were not significantly affected by demographic variables (*p* > 0.05; F1 LD F1). In the analyses performed separately at stations 2 and 3, those who were female, weighed under 70 kg, and were shorter than 170 cm had longer estimation durations, which was statistically significant (*p* < 0.05; F1 LD F1) (Table 7).

**Table 7:** Differences in primary outcome measures between dichotomous demographic groups—gender (female vs. male), weight (< 70 kg vs. ≥70 kg), and height (< 170 cm vs. ≥170 cm)—were examined among all users of the MAR method (Group 1 performed MAR in period II, n_1_ = 70, plus group 2 performed MAR in period I, n_2_ = 70).

*N* = 140			Demographic variables		*n*	Estimated volume (ml)							Estimation duration (sec)						
						Median (IQR)		Mean (SD)		95% CI	p*		Median (IQR)		Mean (SD)	95% CI		p*	
MAR method	STATION1		♂♀	Female	78	100 (80–120)		105.3 (29.5)		98.7–112	0.6		9.0 (5.9–12)		10.1 (5.8)	8.8–11.4		0.46	
				Male	62	100 (80–120)		103.9 (27.5)		96.9-110.9			8.3 (6.3–10.7)		8.7 (2.9)	7.9–9.4			
			kg	< 70 kg	90	100 (90–120)		106.7 (27.7)		100.9-112.5	0.14		8.7 (6.0-11.7)		9.8 (5.5)	8.7–10.9		0.87	
				≥ 70 kg	50	100 (80–120)		101.2 (29.9)		92.6-109.7			8.5 (6.3–11)		8.9 (3.1)	8.0-9.7			
			cm	< 170 cm	63	110 (80–130)		107.9 (30.1)		100.2-115.5	0.13		8.7 (5.9–12.4)		10.2 (6.1)	8.7–11.8		0.71	
				≥ 170 cm	77	100 (80–120)		102.1 (27.2)		95.9-108.3			8.6 (10.9–6.2)		8.9 (3.2)	8.2–9.6			
	STATION2		♂♀	Female	78	260 (200–285)		265.6 (84.5)		246.6-284.7	0.051		14.5 (10.9–21.1)		16.3 (7.2)	14.7–17.9		0.002	
				Male	62	240 (191–260)		243.2 (78.3)		223.8–263.0			11.7 (9.5–15.6)		12.9 (5.1)	11.6–14.2			
			kg	< 70 kg	90	260 (200–285)		260.4 (82.1)		243.1-277.6	0.2		14 (10.6–20.1)		15.6 (6.8)	14.2–17.0		0.028	
				≥ 70 kg	50	240 (200–260)		247.2 (82.7)		223.7-270.7			11.8 (9.6–15.6)		13.3 (5.9)	11.7–15.0			
			cm	< 170 cm	63	260 (200–300)		266.2 (90.9)		243.3-289.1	0.23		15.1 (20.9–11.3)		15.9 (6.3)	14.3–17.5		0.01	
				≥ 170 cm	77	250 (200–280)		247.1 (73.9)		230.3-263.9			12 (9.5–16.4)		13.8 (6.6)	12.3–15.3			
	STATION3		♂♀	Female	78	640 (500–805)		709.1 (298.3)		641.9-776.4	0.096		40.1 (54-25.1)		39.5 (15.3)	36.0-42.9		0.001	
				Male	62	550 (350–800)		642.6 (269.1)		574.2-710.9			27.1 (21.6–39.2)		31.1 (14.0)	27.8–34.7			
			kg	< 70 kg	90	600 (500–800)		688.3 (293.3)		626.9-749.8	0.53		36.6 (24-51.4)		37.8 (15.3)	34.6–40.9		0.049	
				≥ 70 kg	50	590 (450–850)		664.0 (276.5)		585.4-742.6			30.3 (21.5–40.5)		32.2 (14.8)	28.0-36.4			
			cm	< 170 cm	63	640 (500–840)		718.7 (316.1)		639.1-798.3	0.17		40.8 (54 − 25)		39.5 (15.5)	35.6–43.4		0.009	
				≥ 170 cm	77	580 (460–800)		647.7 (257.8)		589.1-706.2			30.2 (22.5–41.3)		32.7 (14.5)	29.4–36.0			
F1 LD F1 Tstat-df-p		Gender 2.432 1.000 Station 1818.168 1.725 Gender x Station 0.840 1.725					p (0.119) p (< 0.001) p (0.417)		Weight 1.444 1.000 Station 1835.885. 1.718 Weight x Station 0.445 1.718			p (0.229) p (< 0.001) p (0.611)		Height 2.342 1.000 Station 1627.335 1.722 Height x Station 0.096 1.722			p (0.126) p (< 0.001) p (0.882)		

For these analyses, P* values were determined using F1 LD F1 model via the *NparLD* package in the R programming language. Pairwise comparisons within each station were performed using the Bonferroni Correction. Data were considered statistically significant at (*p* < 0.05). MAR method: Merlin, Alter, Raffel method. DV: Demographic Variables SD: Standard Deviation. IQR: Interquartile Range. Tstat-df-p: Test statistic-degrees of freedom-p value.

## Discussion

### Limitations and strengths

The present study had several limitations and strengths. One limitation was associated with the MAR method, which is effective only on selected non-absorbent surfaces^10^. Consequently, the study was conducted exclusively on flat, non-absorbent surfaces. Furthermore, the study did not include estimates for all potential blood loss volumes. A 150 ml scenario was added to the existing scenarios of 75 ml and 750 ml from the study by Merlin et al., which limited the generalizability of the findings. The quality indicators related to the MAR method (such as ideal application time and ideal fist height from the ground) are not yet clear. Therefore, the participants’ practices could not be standardized. Finally, the inclusion of only clinically inexperienced students as participants in the study provided valuable insights into the use of the MAR method as a clinical education tool; however, it limited the generalizability of the findings to the clinical practice of experienced professionals.

The primary strength of this study lies in its randomized controlled trial design, which enhances the reliability of the findings. Unlike the study by Merlin et al., this research analyzed both blood loss volume estimates and the time required for these estimations, addressing previously identified limitations. Furthermore, it explored the relationship between MAR estimates and physical variables such as gender, height, and weight, offering a more comprehensive understanding of the factors influencing estimation accuracy.

### Interpretation

According to previous studies^2–11^, healthcare students and professionals have struggled to accurately predict external blood loss using the VE method due to its inherent limitations. Consistent with findings in the literature, the present study demonstrated that the VE method tended to overestimate simulated blood volumes in small (Station 1) and medium (Station 2) blood pools, while underestimating them in large blood pools (Station 3)^2,3,7,15,16^. Additionally, the VE method displayed higher standard deviations and more extreme minimum and maximum values, again in line with the literature^10,19,20^. In our study, both the extreme estimation values and the standard deviations obtained using the VE method were higher, consistent with findings in the existing literature.

In this study, the standard deviations of mean and extreme blood loss estimates with the MAR method decreased at Station 3 by 51% (intergroup) and 59% (intragroup); at Station 2 by 59% and 74%; and at Station 1 by 76% and 80%, respectively. These reductions were classified as moderate for Stations 2 and 3, and as maximum for Station 1. These findings were also consistent with the study by Merlin et al., in which they proposed the MAR method^10^. These results support Hypothesis 1 of this study. In intragroup analyses based on successive VE and MAR estimates from the same participants, the reduction observed in extreme estimates was greater than that in intergroup analyses based on single-method estimates from different participants. Similar to pre-post study designs, this may reflect a method effect; however, the potential impact of repeated measurements should not be overlooked. Therefore, the F1 LD F1 model, a nonparametric alternative to Repeated Measures Mixed ANOVA, was used for intergroup analyses, and the F2 LD F1 model, a nonparametric equivalent of Repeated Measures Two-way ANOVA, was applied for intragroup analyses, taking into account the repeated measures structure.

From a statistical perspective, a higher standard deviation indicates greater dispersion around the mean and reflects inconsistency in estimates across clinically comparable cases. The presence of extreme values further underscores the susceptibility of the VE method to both underestimation and overestimation errors. These findings suggest that the VE method exhibits considerable variability and reduced accuracy in quantifying blood loss during external bleeding.

Clinically, such variability has significant implications for the reliability and practical utility of the VE method in real-world scenarios. Accurate estimation of blood loss is essential for guiding timely and appropriate clinical interventions, particularly in emergency care, prehospital settings, trauma management, and surgical procedures. Overestimation may result in unnecessary interventions—such as fluid resuscitation or blood transfusion—whereas underestimation can delay critical treatment, increasing the risk of hypovolemic shock and other adverse outcomes. Therefore, the wide range and high variability inherent in VE outputs undermine its reliability as a standalone tool in clinical decision-making. These findings emphasize the need to complement VE with more objective, standardized, and validated methods when accurate blood loss assessment is required. On the other hand, the lower standard deviations and reduced number of outliers associated with the MAR method indicate that it may serve as a more reliable and consistent tool than the VE method for use in clinical settings.

When considering raw estimations of blood loss, there were no statistically significant differences between the two methods in either intergroup or intragroup analyses. However, based on error metrics such as mean difference, median differences, and percent error in Tables 5 ad 6, the MAR method enabled participants to estimate actual blood volumes in the stations more accurately. Improvements in estimations were evidenced by a reduction in differences (errors) relative to the actual blood volumes, and this was statistically significant. Furthermore, the MAR method minimized extreme values of blood loss estimates by reducing the interquartile ranges and standard deviations of differences (errors), thereby improving precision. The MAR method demonstrated greater accuracy in blood loss estimations than the VE method, regardless of whether the analysis was intergroup or intragroup, particularly on flat and nonabsorbent surfaces. These results support Hypothesis 1 of this study. These findings were also consistent with the study by Merlin et al.^10^.

Larger volumes, in particular, appear to cause greater estimation errors. At Station 3, where a volume of 750 ml was simulated, both the MAR and VE methods showed a tendency to underestimate the actual blood loss. The underestimation of higher blood loss volumes can be related to the difficulties inherent in both methods.

In the Visual Estimation (VE) method, large blood pools may cause perceptual saturation, making the volume appear smaller. Uniform color and blurred edges reduce contrast, and the absence of a reference object further impairs accuracy^21^. In the MAR method, the algorithm may have difficulty segmenting large, homogeneous blood areas, especially with unclear edges or low contrast^10^. Additionally, at higher volumes, blood may pool vertically, which the MAR method—based on two-dimensional surface area—does not capture, leading to further underestimation^10^.

To improve VE accuracy, training with reference images showing various volumes has been shown to significantly improve visual estimation skills^22^. The use of standardized reference objects as calibration tools (e.g., hand, paper, or ruler) also enhances estimation reliability^23^. In our study, the VE method served as the control group, whereas the MAR method, which utilizes the fist as a reference object, was designated as the experimental group.

For the MAR method, improved training and standardization are recommended to enhance its application and improve its accuracy^10^. Educational materials featuring varied blood loss scenarios across different surfaces can reduce user error. Furthermore, since blood behaves differently depending on the material (e.g., fabric, vinyl, wood), surface-specific calibration models that account for these properties could improve accuracy^24^. The estimation of blood loss should consider the non-linear behavior of fluid spreading^24^. As the volume of blood increases, the surface area covered does not expand proportionally. Beyond a certain threshold, blood begins to accumulate in depth rather than spreading uniformly, resulting in more prominent blood pooling rather than extended coverage. Moreover, surface-related factors—such as slope, texture, and absorbency—further influence the spreading pattern. The same amount of blood may form pools of different sizes (larger or smaller) depending on the characteristics of the surface. This variability highlights the importance of surface calibration and contextual interpretation in all visual, manual (like the MAR method), or algorithm-based blood loss estimation methods^24^.

In both intergroup and intragroup analyses, median estimation durations at Station 1 were similar for both methods. However, at Stations 2 and 3—where simulated blood loss was higher—the MAR method significantly increased estimation durations. Specifically, the mean estimation time with the MAR method increased by 270% (intergroup) and 290% (intragroup) at Station 3, by 87% and 96% at Station 2, and showed a slight change at Station 1 (a 3% decrease and 5% increase), respectively. Especially at Stations 2 and 3, the increases were classified as the maximum. The MAR method was associated with significantly longer estimation durations as compared with those using the VE method. The findings related to estimation durations supported this study’s Hypothesis 2. In the literature, no data could be found regarding the blood loss estimation times for either the VE method or the MAR method.

The MAR method required a longer estimation time, particularly in the high-volume scenario at Station 3. This may limit its practicality in time-sensitive clinical environments. While the MAR method enhances the accuracy of blood loss estimation by reducing estimation errors, outliers, and standard deviations, its time-related burden should be considered in the context of a trade-off analysis.

At Station 3, the average estimation time was approximately 34 s. In prehospital settings—especially during the management of patients in or at risk of hypovolemic shock, where rapid decision-making and intervention are critical—an estimation period of half a minute or longer may negatively impact clinical outcomes. Nevertheless, there appear to be no faster and more accurate alternatives to the MAR method in prehospital environments, and existing portable measurement devices may not be cost-effective for many institutions.

In hospital settings, methods such as venous blood gas analysis and complete blood count are frequently used alongside visual estimation to assess blood loss. However, despite variability between institutions, the average turnaround times for venous blood gas and complete blood count are typically 30 min^25^. Therefore, the MAR method may serve as a practical interim solution for estimating blood loss until more definitive laboratory results become available.

Merlin et al.^10^ predicted that demographic characteristics would affect blood loss estimations using the MAR method because it was developed based on males weighing over 70 kg and taller than 170 cm. Contrary to this prediction, gender, height, and weight variables did not affect the quantitative estimations using the MAR method in the present study. These findings did not support Hypothesis 3 in this study. In this study, median estimation durations using the MAR method at Station 1 did not differ significantly by demographic variables. However, at Stations 2 and 3, durations were significantly longer—by 23% and 47%, 19% and 21%, and 26% and 35%—for participants who were female, weighed less than 70 kg, and were shorter than 170 cm. These findings supported Hypothesis 4. These differences, observed as an increase in estimation durations, may be explained by the possibility that individuals with smaller body size and smaller fists needed more time to estimate larger blood volumes using the MAR method. However, the clinical significance of these differences remains unclear. No data were found in the literature on the relationship between blood loss estimates obtained using the MAR method and demographic variables such as gender, height, and weight.

Previous reports showed that training programs improved the ability of doctors and paramedics to predict visually the amount of external blood loss^11,26–29^. Therefore, incorporating basic MAR method training into clinical education and standardizing all aspects of its instruction appears to be valuable for both healthcare students and professionals. On the other hand, the inclusion of only clinically inexperienced students as participants in the MAR method’s clinical education study limits the generalizability of the findings regarding the MAR method to clinical practice. While the results offer valuable insights into the method’s applicability, predictive accuracy among novices, and using as a clinical skill within undergraduate medical and paramedic education, they should be interpreted within the context of a simulated environment and a non-clinician population.

Clinical settings are typically high-pressure and unpredictable. While experienced professionals are more likely to maintain consistent diagnostic reasoning and effective time management under stress, students may demonstrate greater variability in performance or experience cognitive overload. These differences should be taken into account, as they may lead to divergent outcomes when the MAR method is applied by trained clinicians in real-world conditions. However, in our reference study conducted by Merlin et al.^10^, the participants were healthcare professionals with an average of 10 years of combined training and clinical experience, and the findings were consistent with the primary results of our study, which was conducted with medical and paramedic students, and supported the effectiveness of the MAR method.

To enhance the external validity of the MAR method, future research should involve healthcare professionals with varying levels of clinical experience. Its application should be assessed in real clinical environments, such as emergency departments or prehospital care settings, with a focus on real patient management and patient-centered outcomes^10,30^.

Consequently, it seems worthwhile to identify departmental strategies in various health fields that could foster the MAR method’s ability to improve estimation accuracy as part of ongoing training programs and, if necessary, to formulate appropriate training programs (learning objectives, teaching techniques, training, and assessment). Nevertheless, validation of the MAR method under real-time, high-pressure conditions would provide a more robust understanding of its clinical utility, feasibility, and limitations in everyday practice.

## Conclusions

The study found that the MAR method significantly improved the accuracy of external blood loss estimation by reducing differences (errors) from actual blood volume, interquartile ranges, standard deviations of errors, and percentage errors. However, although the MAR method improved estimation accuracy, it also resulted in an extended estimation time. Additionally, estimation volumes were not influenced by variables such as gender, height, or weight, but estimation durations were significantly impacted by these factors.

Accurately estimating the amount of external blood loss is considered a crucial skill, as it is essential for the management of hemorrhagic shock by professionals in emergency settings. To improve the accuracy of blood loss estimation, formal training is indispensable for medical and paramedic students. Incorporating the MAR method into structured clinical skills training within both undergraduate and postgraduate programs in emergency medicine and paramedic education may be beneficial in the context of effective external blood loss management. To further increase the external validity of the MAR method, future studies should involve healthcare professionals, real patient scenarios, and patient-centered outcome measures.

## Supplementary Information

Below is the link to the electronic supplementary material.

Supplementary Material 1

Supplementary Material 2

## Data Availability

The data that support the findings of this study are available from the corresponding author upon reasonable request.
