# Supplementary material for: The MAR method versus the visual estimation method in predicting external blood loss: a randomized controlled study
Source: Sci Rep. 2025 Aug 24;15:31091. doi: 10.1038/s41598-025-16169-0 (PMC12375695; doi:10.1038/s41598-025-16169-0)

|  |  | Estimated volume (ml) | | |  |  | | Estimation duration (sec) | | | |
| --- | --- | --- | --- | --- | --- | --- | --- | --- | --- | --- | --- |
| Sitation | **Statistics** | **VE Method p^*^** | | **MAR Method** | **p^*^** | **VE Method** | **p^*^** | | **MAR Method** | **p^*^** |  |
| Sitation 1  (75 ml) | Median (Range) | 145 (10-500) | <0.001 | 100 (50-200) | 0.028 | 7.4 (1.1-37.4) | 0.006 | | 8.6 (3.3-20.2) | 0.055 | |
|  | Mean (SD) | 157.3 (130.3) |  | 106.6 (31.2) |  | 9.4 (6.8) |  |  | 9.1 (3.9) |  |  |
|  | Skewness | 0,9 |  | 0.3 |  | 2.1 |  |  | 0.9 |  |  |
| Sitation 2  (150ml) | Median (Range) | 200 (10-1100) | <0.001 | 240 (120-640) | <0.001 | 6.6 (1.5-28.4) | <0.001 | | 13.5 (3.6-32.7) | 0.023 | |
|  | Mean (SD) | 273.1 (241) |  | 255.2 (98.3) |  | 7.7 (5.2) |  |  | 14.4 (6.5) |  |  |
|  | Skewness | 1.4 |  | 1.7 |  | 1.9 |  |  | 0.80 |  |  |
| Sitation 3  (750 ml) | Median (Range) | 525 (15-3000) | <0.001 | 600 (290-1700) | 0.005 | 7.4 (1-36.8) | <0.001 | | 31.1 (5.1-67) | 0.028 | |
|  | Mean (SD) | 707.5 (638.2) |  | 692.1 (312.4) |  | 9.4 (6.9) |  |  | 34.8 (15.9) |  |  |
|  | Skewness | 1.66 |  | 1.1 |  | 1.8 |  |  | 0.35 |  |  |

**Table 1.** Evaluating the normality assumption**.** p^*^ values determined by Kolmogorov-Simirnov normality test: <0.001 indicates p-values ​​less than 0.001.

**Q-Q PLOTS for the MAR vs VE Study**

The Kolmogorov–Smirnov test indicates that most variables violate the normality assumption. For all three volume conditions (75 ml, 150 ml, and 750 ml), both VE and MAR volume estimates (ml) and prediction times (s) yielded p-values below 0.05, leading to rejection of the normality assumption. The only borderline case is the MAR prediction time at 75 ml (p = 0.055). Pronounced deviations in the Q–Q plots, especially in the tails, further support these findings. Given that the data are largely non-normal, non-parametric tests were used in this study.

The Q–Q plots are presented below:

**Volume estimations (ml) for Station 1**

**
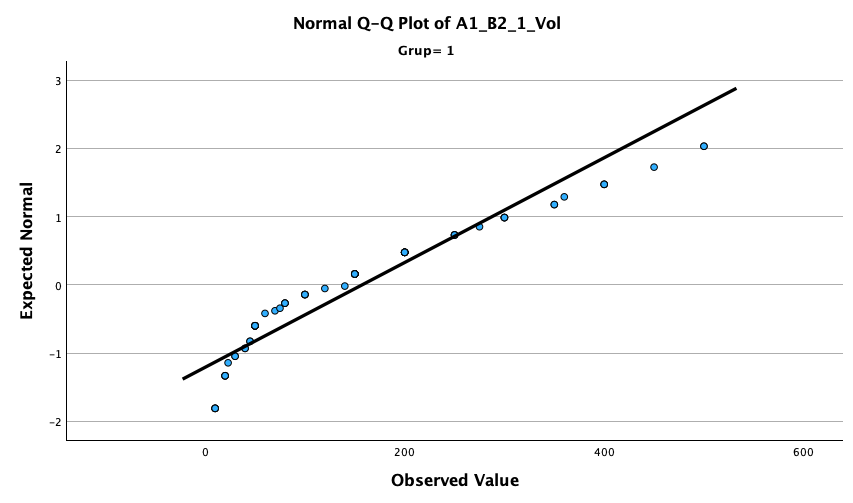

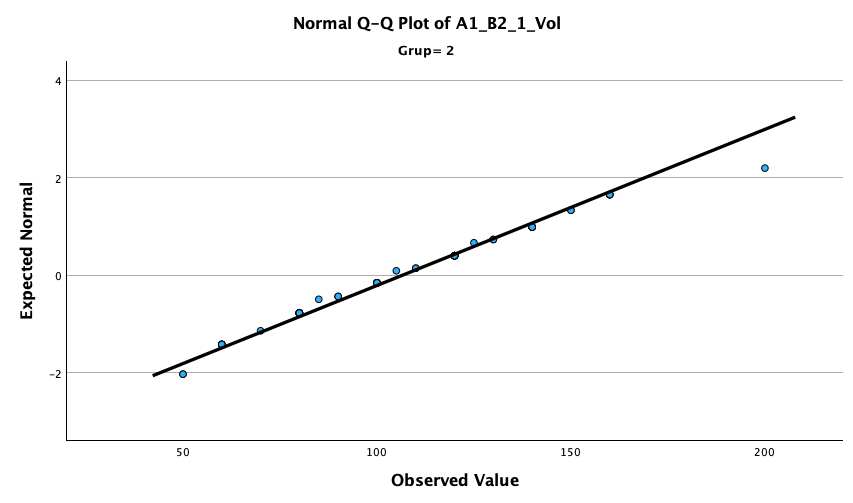
**

**Volume estimations (ml) for Station 2**


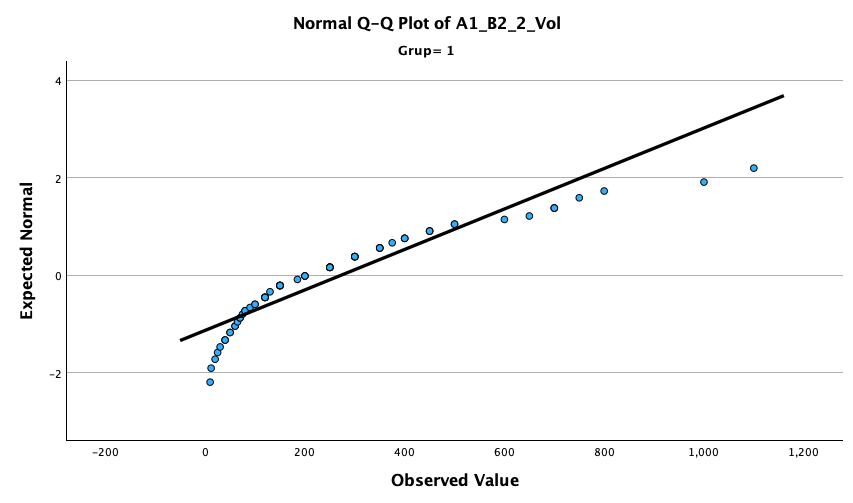

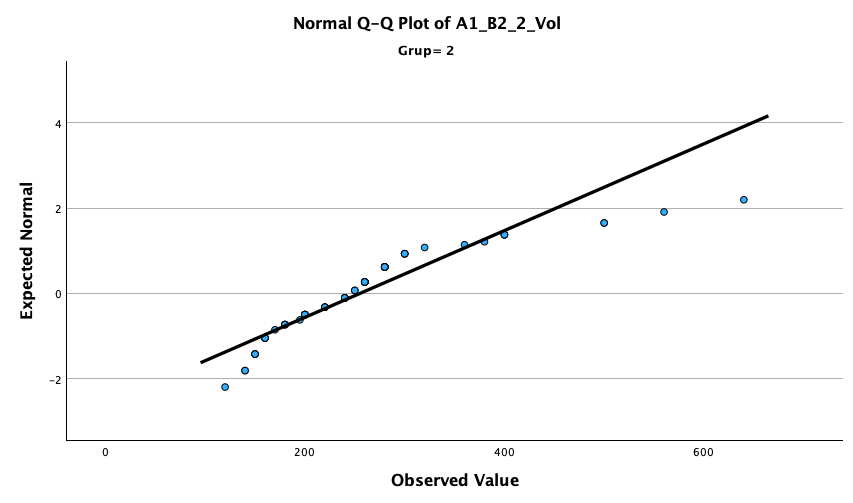


**Volume estimations (ml) for Station 3**


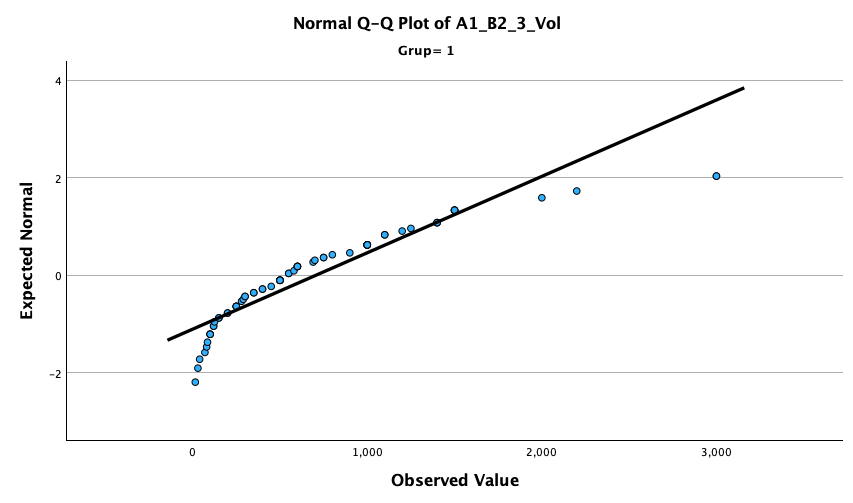

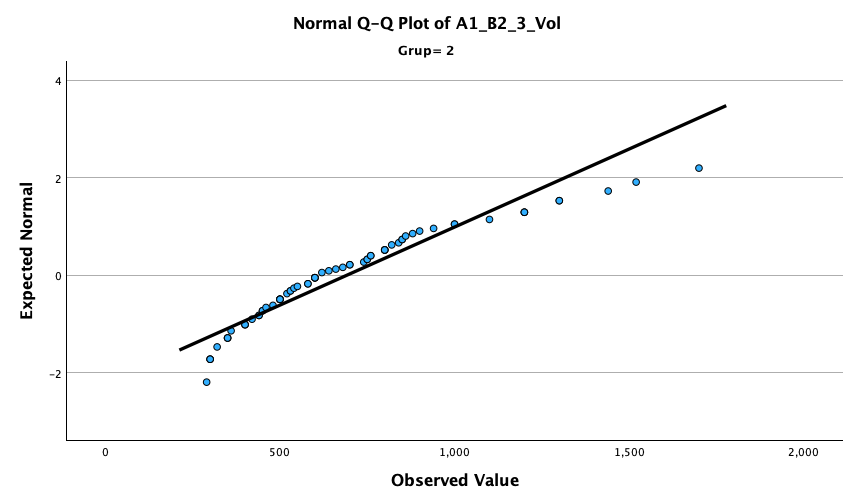


**Estimation Durations (sec) for Station 1**


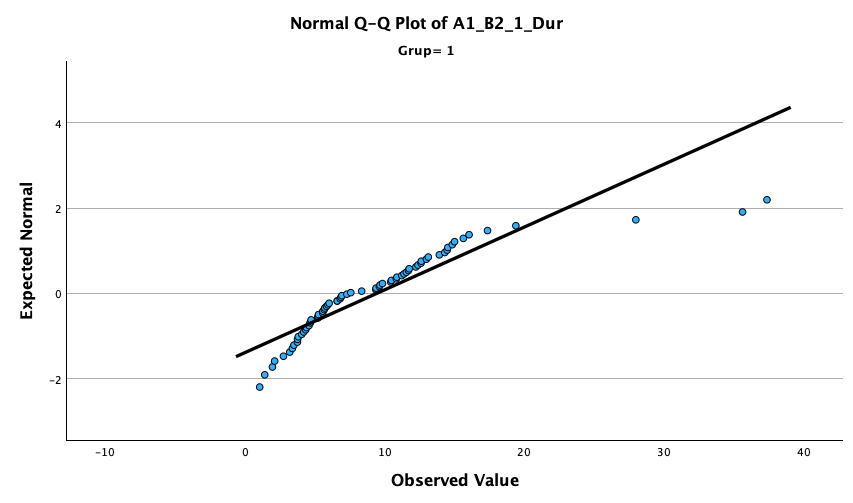

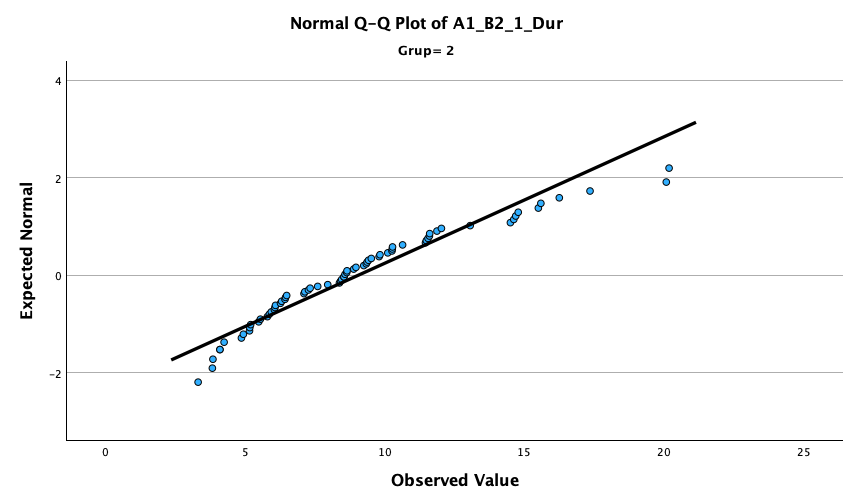


**Estimation Durations (sec) for Station 2**


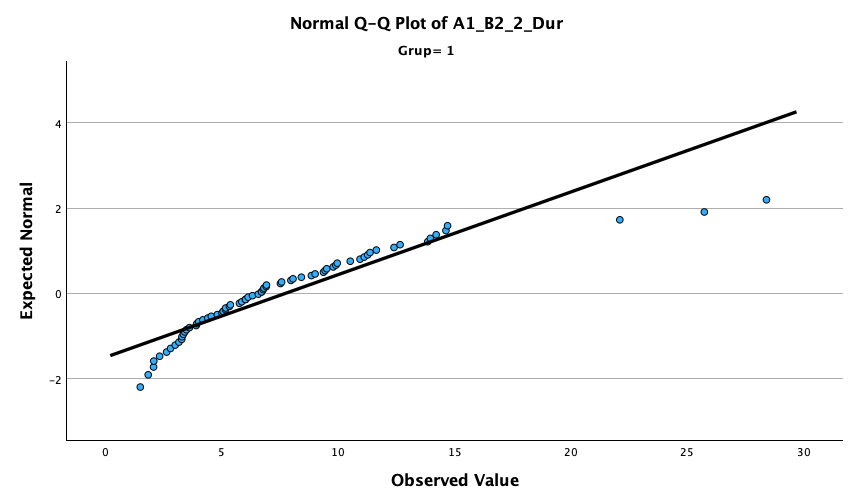

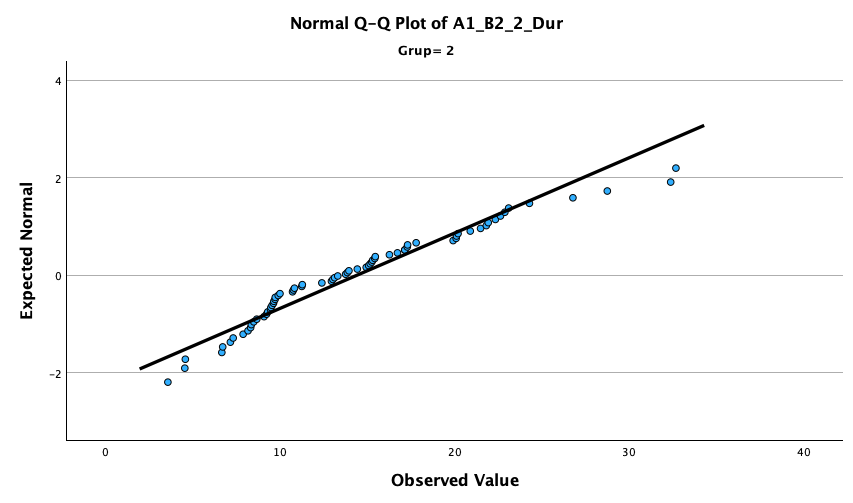


**Estimation Durations (sec) for Station 3**


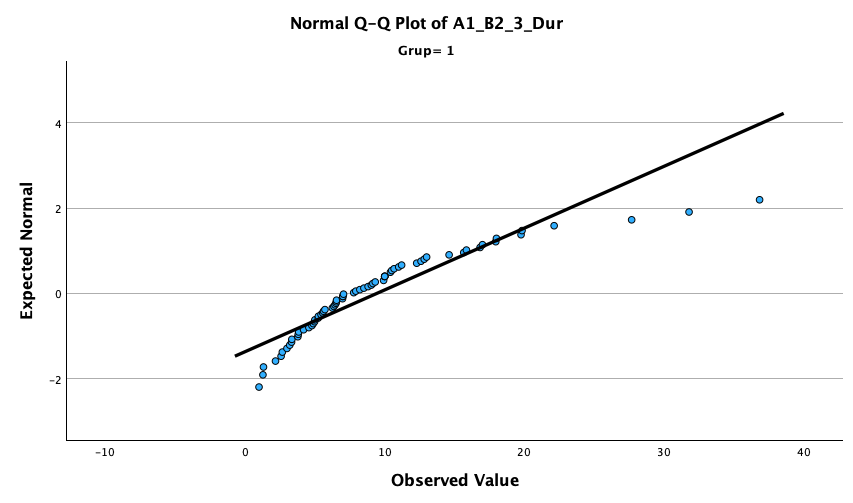

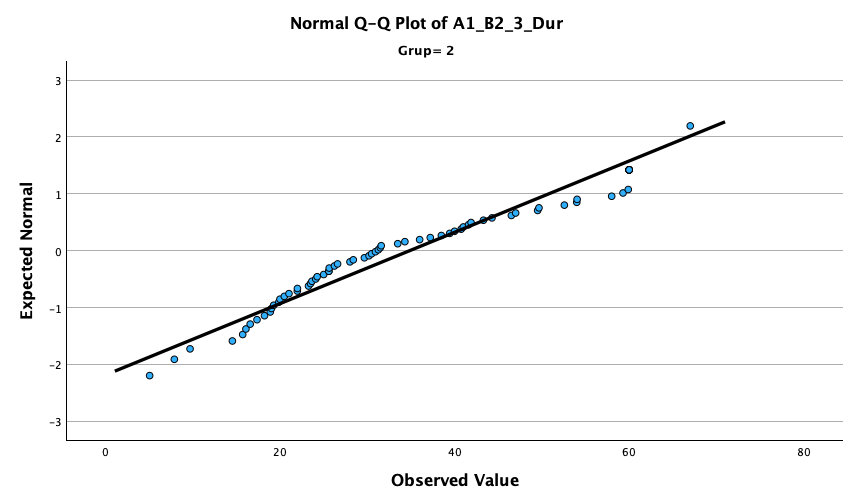

Supplement: Supplementary file 2 — Supplementary Material 2 [file 41598_2025_16169_MOESM2_ESM.docx]
